# Neoangiogenesis in early cervical cancer: Correlation between color Doppler findings and risk factors. A prospective observational study

**DOI:** 10.1186/1477-7819-6-126

**Published:** 2008-11-25

**Authors:** Matias Jurado, Rosendo Galván, Rafael Martinez-Monge, Jesús Mazaira, Juan Luis Alcazar

**Affiliations:** 1 Department of Gynecology, Clínica Universitaria de Navarra, School of Medicine, University of Navarra. Pamplona. Spain; 2 Department of Radiation Oncology, Clínica Universitaria de Navarra, School of Medicine, University of Navarra. Pamplona. Spain

## Abstract

**Background:**

The aim of the present article was to evaluate whether angiogenic parameters as assessed by transvaginal color Doppler ultrasound (TVCD) may predict those prognostic factors related to recurrence.

**Methods:**

A total of 27 patients (mean age: 51.3 years, range: 29 to 85) with histologically proven early stage invasive cervical cancer were evaluated by TVCD prior to surgery. Subjective assessment of the amount of vessels within the tumor (scanty-moderate or abundant) and pulsatility index (PI) were recorded. All patients underwent radical hysterectomy and pelvic lymph node dissection. Postoperative treatment (RT or chemoradiotherapy) was given according to risk factors (positive lymph nodes, parametrial and vaginal margin involvement, depth stromal invasion, lymph-vascular space involvement)

**Results:**

Tumors with "abundant" vascularization were significantly associated with pelvic lymph node metastases, depth stromal invasion > 10 mm, lymph-vascular space involvement, tumor diameter > 17.5 mm, and parametrial involvement. Postoperative treatment was significantly more frequent in patients with "abundant" vascularization (OR: 20.8, 95% CIs: 2 to 211). The presence of scanty-moderate vascularization with a PI < 0.82 or abundant vascularization with either PI > 0.82 or PI < 0.82 was associated with high-risk group in 94.4% of the cases (OR: 21.2, 95% CI: 1.9 to 236.0)

**Conclusion:**

The results are consistent with a relationship between tumor angiogenesis and prognostic factors for recurrence in early cervical cancer. "Abundant" vascularization and PI < 0.82 may be related to postoperative treatment due to risk factors.

## Background

Angiogenesis has gained much attention in oncology in recent years. It has been shown to be an essential event for tumor growth and metastases [1]. Several studies have demonstrated that tumor angiogenesis is an independent prognostic factor in cervical cancer [2-4]. Therefore, the assessment of this factor would seem to be important when evaluating patients with this disease. However, tumor angiogenesis can only be assessed on the surgical specimen after surgery and therefore its prospective use, as part of the treatment plan is difficult.

Transvaginal Color-Doppler Ultrasound (TVCD) allows an *in vivo *non-invasive and prospective assessment of tumor vascularization [5]. Some studies have shown that color and Power-Doppler sonography can be used to depict flow within arterioles and venules > 100 μm [6]. Furthermore, recent developments in this field have enabled depiction of microvasculature (<7–10 μm)[7].

Treatment of early cervical cancer (FIGO stage Ia2, Ib1 and II a <4 cm) is radical hysterectomy (RH) and pelvic lymphadenectomy (PLND). Radiotherapy is equally effective with similar 5-year survival [8]. Some studies have found that local recurrence in early cervical cancer surgically treated is related to several prognostic factors such as tumor size, lymph node (LN) metastases, parametrial or vaginal margins involvement, depth of stromal invasion (DSI) and lymph-vascular space invasion (LVSI). According to these data and based on different patterns of recurrence it has been proposed three different risk groups: low (absence of any risk factor), intermediate (DSI ≥ 10 mm, LVSI), and high risk (LN metastases, parametrial invasion, or vaginal margin invasion) [9-14]. Prospective randomized trials have shown a survival benefit after radiation therapy for the intermediate risk group [15] as well as for the high risk group after concomitant chemoradiation [16]. Nonetheless, patients requiring adjuvant radiotherapy after radical surgery have a higher long-term urologic morbidity as well as intestinal and lymph-vascular complications [17].

The aim of this prospective study is to evaluate whether angiogenesis parameters as assessed by TVCD (amount of intratumoral vessels and blood flow) may predict those prognostic factors related to recurrence. A second objective is to study its ability to predict the need of postoperative treatment.

## Patients and methods

This is a prospective observational study. Clinical, sonographic, and histopathologic data on 27 patients (mean age: 51.3 years, ranging from 29 to 85 years) with histologically proven invasive cervical cancer without evidence of extra-uterine disease by CT scan or MRI, treated at our institution were analyzed. Patients' characteristics are shown in Table 1.

**Table 1:** Patients' characteristics

	**n**	**%**
**FIGO Stage**		
Ia2	1	3.7
Ib1	25	92.6
IIa	1	3.7
Tumor size (cm)*	2.2	(1–3.9)

**Histology**		
SCC	18	66.7
Non-SCC	9	33.3
Grade 1	11	40.7
Grade 2	13	48.1
Grade 3	3	11.1

**Surgery**		
RH-II	22	81.5
RH-III	5	18.5
PLND**	14	(4–37)
+	5	18.5
--	22	81.4
DSI (mm) < 10	8	29.6
DSI > 10 mm	19	70.4
LVSI+	9	33.3
LVSI-	17	62.9
LVSI Unknown	1	3.7

**Postop. Treat**.		
EPRT + Brachitherapy	11	40.7
Chemoradiation	7	25.9
No	9	33.3

*mean, range in parentheses ** median, range in parentheses

SCC = Squamous cell carcinoma. RH = radical hysterectomy. PLND = Pelvic Lymph node dissection. DSI = Depth stromal invasion. LVSI = Lymph-vascualr space invasion. EPRT = External pelvic radiation

All patients underwent TVCD after diagnosis within one week before surgery. Approval of Institutional Review Board approval was obtained. TVCD data was not used for clinical management decisions.

Transvaginal color Doppler sonography was performed in all patients using a Toshiba SSA-370 A (Toshiba Medical Systems, Tokyo, Japan), Sonoace 9900 (Kretztechnik, Zipf, Austria) or Voluson 730 (GE, Milwaukee, USA) machines equipped with real-time 5–7 MHz sector electronic array endovaginal probes with 5.0 MHz pulsed and color Doppler capabilities.

After the endovaginal probe was gently inserted into the vagina, the uterus and adnexal regions were scanned. Cervical tumor size was estimated using electronic calipers on the screen.

**Figure 1:**
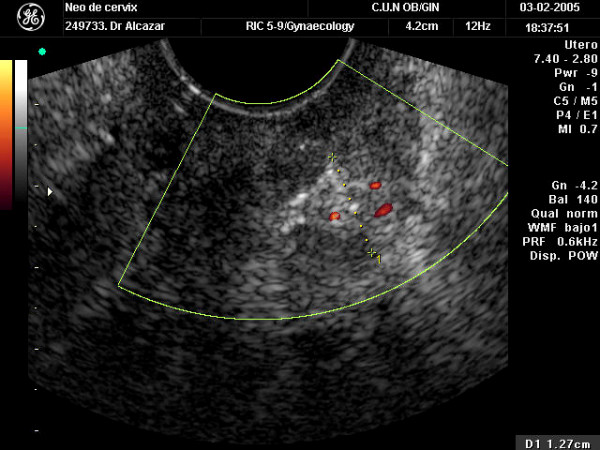
Transvaginal color Doppler ultrasound showing a cervical cancer with scanty vascularization.

**Figure 2:**
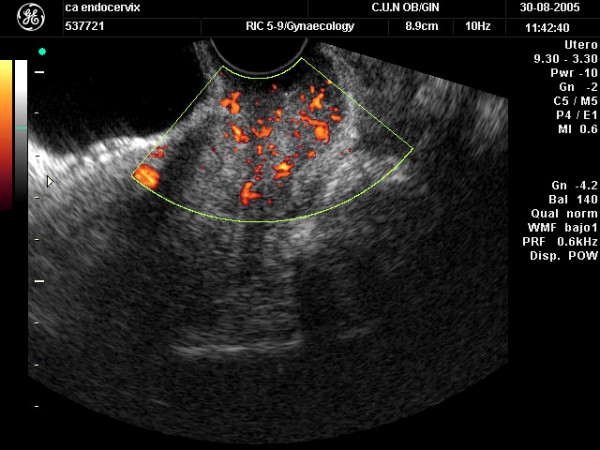
Transvaginal color Doppler ultrasound showing a cervical cancer with abundant vascularization.

After tumor size was estimated, color Doppler gate was activated to identify intratumoral vessels. Color sensitivity was set for slow velocities (1.5–10 cm/sec. PRF was set at 6.0 kHz). Color gain was set at maximum level and then lowered until noise disappeared. As peripheral vessels could not be reliably ascertained as neovascularized or pre-existing vessels only central vessels were evaluated. We arbitrarily considered as "central vessels" those located at least at 5 mm far from the tumor's border. The amount of vascularization was subjectively stated as scanty/moderate (only few color spots seen) or abundant (multiple color spots seen) (Figures 1 and 2). After a vessel was identified, pulsed Doppler volume sample was activated to obtain the flow velocity waveform (FVW). Pulsatility index (PI = [maximum peak systolic velocity- end diastolic velocity]/mean velocity) was automatically calculated for each vessel. We chose PI arbitrarily. The lowest PI found was taken for analysis.

All sonographic examinations were performed by one of the authors (JLA). Intra-observer coefficient of variation (CV) for tumor size and PI were 5%, and 6%, respectively. CV was calculated by performing two different measurements at 10-minute interval in the first five patients

Following our institution's guidelines, surgical treatment was a type II or III RH with PLND. Patients with two or more intermediate risk factors received further treatment with external pelvic radiation (EPRT) (45 Gy) and vaginal high dose brachytherapy (HDB) (10 to 20 Gy). For patients with at least one high risk factor the same radiation regimen with concomitant weekly chemotherapy with Taxol 50 mg/m^2 ^and Cisplatinum 40 mg/m^2 ^for a total number of five courses was provided.

The Kolmogorov-Smirnov test was used to assess normal distribution of continuous variables. One way analysis of variance with Bonferroni post-hoc or Mann-Whitney tests were used to compare RI and PI according to different prognostic factors. The χ^2 ^with Pearson's correction was used to compare categorical data. Receiver operating characteristics (ROC) curves were plotted to determine the best stromal invasion depth, tumor diameter and lowest PI cutoff values to predict postoperative treatment. Odd Ratios and positive likelihood ratios (LR+) were also determined. Sensitivity, specificity, positive predictive value (PPV) and negative predictive value (NPV) were also calculated.

A p value ≤ 0.05 was considered statistically significant. All statistical analyses were performed using the Statistical Package SPSS 13.0.

## Results

### Prognostic factors prediction

ROC curves showed that the best cut-off values for tumor diameter and DSI for predicting postoperative treatment were 17.5 mm (AUC: 0.66, 95% CI: 0.41 to 0.91) and 10 mm (AUC: 0.78, 95% CI: 0.57 to 0.98), respectively.

The amount of vascularization was significantly associated with prognostic factors: Tumors with "abundant" vascularization were significantly associated with pelvic LN metastases, DSI > 10 mm, LVSI, tumor diameter > 17.5 mm, and parametrial involvement (Table 2). Lowest PI were significantly lower in patients with DSI > 10 mm (Table 3).

**Table 2:** Amount of vascularization and prognostic factors

Parameter		Scanty-Moderate (%)	Abundant (%)	p
PLN	+	0	5 (43)	0.025
	-	13 (100)	9 (57)	

DSI	<10	8 (100)	0	0.001
	>10	5 (26)	14(74)	

LVSI	+	2 (15)	8 (85)	0.021
	-	11 (69)	5(31)	

T. size	< 17.5 mm	7 (78)	2(22)	0.037
	> 17.5 mm	6 (33)	12(67)	

Parametrium	+	0	6(42.9)	0.016
	-	13(100)	8 (57.1)	

Histology	SCC	7 (53.8)	11 (78.6)	0.171
	Non-SCC	6 (46.2)	3 (21.4)	

SCC = Squamous cell carcinoma. PLN = Pelvic Lymph node. DSI = Depth stromal invasion. LVSI = Lymph-vascualr space invasion.

**Table 3:** Pulsatility index and prognostic factors

	Lowest PI*	P value
PLN		0.473
Negative	0.89 (0.68 – 1.10)	
Positive	0.74 (0.52 – 0.95)	

DSI		0.004
< 10 mm	1.20 (0.91 – 1.60)	
> 10 mm	0.74 (0.55 – 0.92)	

LVSI		0.073
Negative	1.00 (0.75 – 1.30)	
Positive	0.68 (0.44 – 0.92)	

Tumor size		0.158
< 17.5 mm	1.06 (0.68 – 1.40)	
> 17.5 mm	0.80 (0.60 – 1.40)	

Parametrium		0.171
Negative	0.95 (0.73 – 1.17)	
Positive	0.67 (0.48 – 0.86)	

Histology		0.406
SCC	0.84 (0.63 – 1.05)	
Non-SCC	0.99 (0.61 – 1.37)	

* Expressed as median, range in parentheses.

SCC = Squamous cell carcinoma. PLN = Pelvic Lymph node. DSI = Depth stromal invasion. LVSI = Lymph-vascualr space invasion.

### Further treatment prediction

Postoperative treatment (RT or chemoradiotherapy) was significantly more frequent in patients with "abundant" vascularization (OR: 20.8, 95% CI: 2 – 211). Thirteen out of 18 patients who needed postoperative therapy had abundant vascularization. Only one out of 9 patients who did not need postoperative therapy had abundant vascularization. Sensitivity, specificity, PPV and NPV for this parameter were 72%, 89%, 93% and 61.5%, respectively.

Lowest PI was significantly lower in patients who needed further treatment (0.79, 95% CI: 0.44 to 1.00) as compared with those who did not (1.10, 95% CI: 0.86 to 1.36) (p = 0.041)

**Figure 3:**
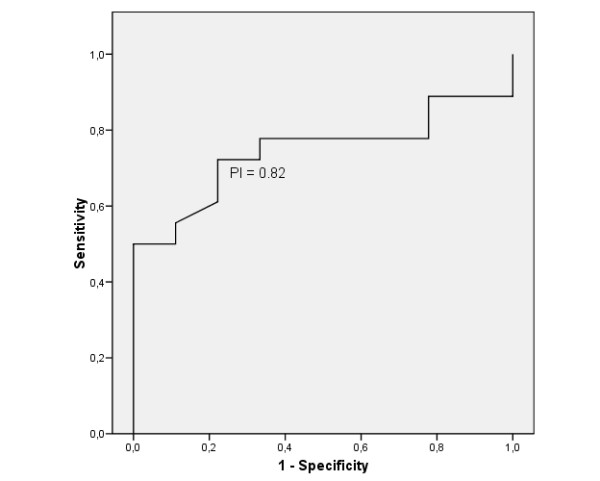
ROC curve for pulsatility index. The best cut-off was 0.82.

ROC curves showed that the best cutoff value for PI was 0.82(AUC = 0.74, 95% CI: 0.56 to 0.93) (Figure 3). Patients with PI < 0.82 needed more frequently postoperative treatment (OR: 9.1, 95% CI: 1.4 to 59.6)

In order to develop a way to predict prospectively patients that would be candidate for postoperative treatment, the combination of the amount of vascularization and PI < 0.82 was evaluated according to prognostic factors. Two main risk groups were established. The high-risk group that was defined as having at least one of the following prognostic factors: LVSI, DSI > 10 mm, tumor size > 17.5 mm, parametrial involvement or LN metastases. The low risk group was defined as not having any of these factors.

The presence of scanty-moderate vascularization with a PI < 0.82 or abundant vascularization with either PI > 0.82 or PI < 0.82 was associated with high-risk group in 94.4% of the cases (OR: 21.2, 95% CI: 1.9 to 236.0) (Table 4). LR+ for these three groups all together was 4.76

**Table 4:** Risk group according to amount of vascularization and PI

	Low Risk	High Risk	Total
Scanty Vascularization and PI > 0.82	5 (55.2%)	4 (44.8%)	9

Scanty vascularization and PI < 0.82 or Abundant vascularization	1 (5.6%)	17 (94.4%)	18

Total	6	21	27

## Discussion

### Prognostic factors prediction

It is generally accepted that the rate of local recurrence for early stage cervical cancer (FIGO Ib1 to II a < 4 cm) is significantly lower than in advanced stages. The presence of LN metastases has an overriding prognostic importance in early stage cervical carcinoma with an overall survival average of 90% if the pelvic nodes are negative and 65% if pelvic nodes are positive. It is also important the number of nodes involved, thus patients with one to three involved nodes reported to have a 72% 5-year survival, whereas the survival of patients with more than three nodes involved averages only 40% [13,18]. Furthermore, based on multivariate analysis, tumor size, LVSI, and depth of cervical stromal invasion are independent predictors of lymph nodes metastases risk and, therefore, disease-free survival [9,13,19,20]. It has also been reported that due to the presence itself of these prognostic factors without pelvic lymph nodes involvement the rate of recurrence may increase from 2% to 31%, mainly locally, after three years [15]. GOG prospective randomized trial [15] has found a statistically significance decrease of local recurrence after radiotherapy in this group of patients. Other prospective randomized trials [16] have found a benefit in overall survival and disease free survival with postoperative concomitant chemoradiation over radiation therapy alone in a higher risk group of patients with early stage and with lymph node metastases, parametrial or vaginal margin invasion due to its mixed recurrent pattern.

Several publications [21-24] have pointed out the capability of transvaginal color-Doppler to assess the intratumoral blood flow in cervical cancer. Velocimetric indexes and color signals correlated with some prognostic factors. Cheng *et al *[25] reported on a group of 35 patients with stage Ib to II cervical cancer in whom they assessed tumor angiogenesis by TVCD. They found that vascular index (VI = number of colored pixels/number of total pixels) correlated with prognostic factors. The higher the VI, the higher the tumor stage, the deeper stromal invasion, the higher the LVSI rate and the higher the pelvic LN metastases rate was. Also interesting was this VI had a good correlation with intratumoral microvessel density as assessed immunohistochemically. The same group reported on a further series of 60 patients with stage Ib to II a but using TVCD. The presence of color signals was associated with a higher probability of LN metastases and parametrial involvement [26].

Hsu *et al *[27] reported their results on 141 patients with early stage cervical cancer in who tumor angiogenesis was assessed by 3-D Power-Doppler. They found that tumor vascularization correlated with tumor volume.

Testa *et al *[28] also found a similar correlation between tumor vascularization and its volume. In our study a significant correlation between prognostic factors and tumor vascularization was found, being the amount of vascularization higher when tumor had deeper stromal invasion, larger diameter, LVSI, parametrial involvement or LN metastases. Vascular flow as assessed by velocimetric indexes (the lowest PI) was correlated only with stromal invasion higher than 10 mm. There was a trend for LVSI. The lack of correlation with the rest of prognostic factors could be due to the small number of patients in this series.

### Postoperative treatment prediction

Cheng *et al *[26] in their above mentioned study performed with TVCD reported results, found that the presence of color signals was associated with a higher probability of LN metastases and parametrial invasion. Although they did not made any specific statistical analysis, they suggested that these findings could be helpful in planning treatment for women with stage I–II a cervical carcinoma.

To the best of our knowledge this is the first study regarding the issue of tumor vascularization and its role to predict further treatment in early cervical cancer treated with radical surgery. We have found that amount of vascularization and the lowest PI found within the tumor were associated with the need for postoperative treatment due to the presence of risk factors. Those with "abundant" vascularization received more frequently adjuvant treatment with radiation with or without simultaneous chemotherapy, especially if PI was < 0.82. However, the clinical use of PI as the unique parameter for predicting further treatment may be questionable because the significant overlapping of individual values observed. This overlapping could be explained by the fact of the small series herein reported.

Another interesting question may be the use of 3D power Doppler vascular indexes. To date the only study reported did not find any relationship between 3D power Doppler indexes and tumor features [28]. In our preliminary experience 3D power Doppler indexes were significantly higher in locally advanced stage tumors as compared with early stage cervical cancer [29]

Over the last ten years much attention has been paid to morbidity after the combination of radical surgery and pelvic radiotherapy. Some publications regarding this issue [8,17] have found a significantly higher risk of postoperative complications, specifically urologic and intestinal. Therefore a judicious pretreatment selection of patients with predictable risk factor for adjuvant therapy would help to select patients who should not be scheduled for primary radical surgery. Whether TVCD and the study of angiogenesis would help to avoid this morbidity as a consequence of a more reasonable plan of treatment based on prospectively predictable prognostic factors needs further evaluation.

With angiogenic parameters, two main groups of risk for adjuvant treatment could be defined. As patients with intermediate risk factors are currently treated with radiation alone [15] and with radiation and simultaneous chemotherapy those with parametrial involvement or LN metastases [16], it will be interesting to define this later subset of patients in a larger series.

## Conclusion

Our results are consistent with a relationship between tumor angiogenesis and prognostic factors for recurrence in early cervical cancer. "Abundant" vascularization and the lowest PI are related to postoperative treatment due to risk factors that can be easily and prospectively assessed by TVCD and these findings encourage following with larger series of study.

## List of abbreviations

TVCD: Transvaginal Color Doppler; PI: Pulsatility index; RT: Radiotherapy; FIGO: Federation International Gynecology and Obstetrics; RH: Radical hysterectomy; PLND: Pelvic lymph node dissection; LN: Lymph node; DSI: Depth stromal invasion; LVSI: Lymph-vascular space invasion; CT: Computed tomography; MRI: Magnetic resonance imaging; EPRT: External pelvic radiation therapy; HDB: High dose brachytherapy; GOG: Gynecologic Oncology Group; OR: Odds ratio; CI: Confidence intervals; ROC: Receiver Operator curves; AUC: Area under the curve; NPV: Negative predictive value; PPV: Positive predictive value; LR: Likelihood ratio; CV: Coefficient of variation.

## Competing interests

The authors declare that they have no competing interests.

## Authors' contributions

JLA was involved in study design, data collection, analysis, patient recruitment and management. MJ was involved in study design, data collection, analysis, patient recruitment and management and preparation of the manuscript. RMM was involved in patient recruitment and management, helped in preparation of draft. RG was involved in data analysis and interpretation of results. The final manuscript was approved by all authors.
